# Unusual Triggering of the Finger Caused by Distal Flexor Tendinopathy: Successful Treatment With Ultrasound-Guided Prolotherapy

**DOI:** 10.7759/cureus.99605

**Published:** 2025-12-19

**Authors:** Yonghyun Yoon, Ji Hyo Hwang, Jaeyoung Lee, Teinny Suryadi, Anwar Suhaimi, Jaehyun Shim, King Hei Stanley Lam

**Affiliations:** 1 Orthopedic Surgery, Hallym University Kangnam Sacred Heart Hospital, Seoul, KOR; 2 Orthopedics, Incheon Terminal Orthopedic Surgery Clinic, Incheon, KOR; 3 Physical Medicine and Rehabilitation, Synergy Clinic, Jakarta, IDN; 4 Physical Medicine and Rehabilitation, Hermina Podomoro Hospital, Jakarta, IDN; 5 Rehabilitation Medicine, Universiti Malaya Medical Centre, Kuala Lumpur, MYS; 6 Rehabilitation Medicine, Universiti Malaya, Kuala Lumpur, MYS; 7 Neurosurgery, Chungdammadi Neurosurgery Clinic, Seoul, KOR; 8 Faculty of Medicine, The Chinese University of Hong Kong, New Territories, HKG; 9 Faculty of Medicine, The University of Hong Kong, Hong Kong, HKG; 10 Board of Clinical Research, The Hong Kong Institute of Musculoskeletal Medicine, Kowloon, HKG

**Keywords:** enthesopathy, insertional tendinopathy, prolotherapy, trigger finger, ultrasound

## Abstract

Trigger finger (TF), traditionally viewed as a stenosing tenosynovitis at the A1 pulley, is commonly treated with corticosteroid injections or pulley release. This case report describes a 61-year-old female with a refractory TF who did not respond to a previous corticosteroid injection. Diagnostic imaging revealed insertional tendinopathy of the flexor digitorum superficialis and flexor digitorum profundus with bony spurs, rather than pathology at the A1 pulley. The patient was successfully treated with two sessions of ultrasound-guided prolotherapy directed at the tendon entheses, resulting in complete resolution of triggering and pain. This case challenges the conventional model and suggests that insertional flexor tendinopathy can be a primary cause of TF, warranting a paradigm shift in diagnosis and treatment toward enthesis-focused approaches.

## Introduction

Trigger finger (TF), or stenosing tenosynovitis, is primarily attributed to a size mismatch between the flexor tendon and the A1 pulley [1]. Consequently, treatment strategies predominantly target the A1 pulley. However, emerging evidence indicates that pathology at other sites, such as the camper’s chiasm, A2 pulley, or the palmar aponeurosis, flexor retinaculum, can also cause triggering [2-6]. Cases of persistent symptoms after A1 pulley release further support this notion.

Diagnosis typically relies on history and physical examination, with ultrasound gaining prominence for evaluating the A1 pulley and volar plate [7,8]. Although recent discussions on TF have expanded to include general tendinopathy [9-11] and pathologies at the Camper’s chiasm, insertional tendinopathy remains a notably overlooked entity and is consistently absent from diagnostic considerations.

First-line treatments for TF include medication, physiotherapy, and splinting, with a reported success rate of almost 70% [12]. For persistent cases, corticosteroid injection (CSI) is recommended [13]. The standard treatment algorithm for refractory cases progresses to A1 pulley release, either percutaneously or via open surgery [14]. However, as data accumulates, complications from these procedures are increasingly reported, ranging from superficial infection and pain to incomplete release requiring re-operation, bowstringing, and complex regional pain syndrome (CRPS) [15-17].

Given these potential complications, alternative treatments such as prolotherapy and hydrodissection have been explored [18,19], but they largely remain within the conventional paradigm of focusing on the A1 pulley and volar plate. Therefore, this case report aims to present a case of TF caused by insertional flexor tendinopathy that was successfully managed with prolotherapy, thereby challenging the conventional paradigm and proposing a significant shift in diagnostic and therapeutic approaches.

## Case presentation

A 61-year-old woman presented with a one-year history of pain and triggering in her left middle finger. Her medical history was significant only for hypothyroidism following a total thyroidectomy, managed with levothyroxine. She had received a CSI consisting of 10 mg of triamcinolone acetonide mixed with 0.5-1 mL of 1% lidocaine one month after symptom onset, which provided only three weeks of temporary relief, followed by a recurrence and a fluctuating course. For the month preceding her visit, she reported morning stiffness, active triggering, a sensation of heat, and tenderness.

**Figure 1:**
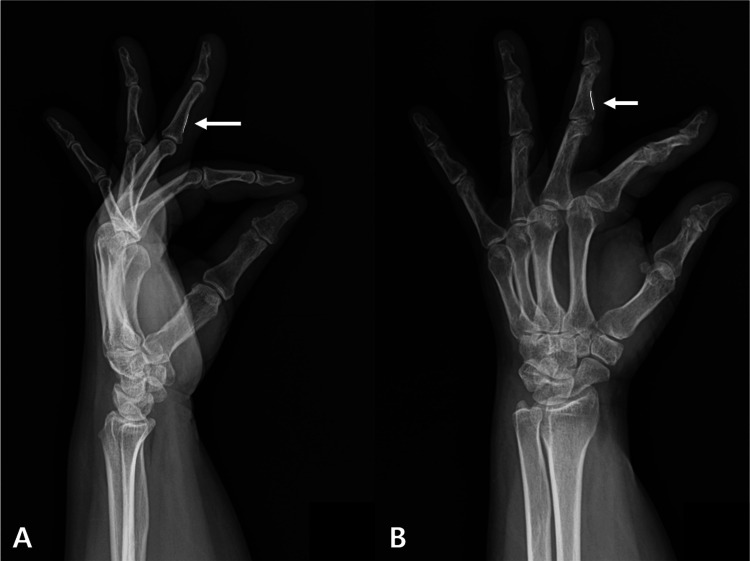
In lateral (A) and oblique (B) X-rays, a bony ridge (white arrow) is observed outside the normal line (white line) of the middle phalanx.

Physical examination revealed tenderness and swelling over the volar aspect of the metacarpophalangeal (MCP) joint, which was locked in flexion. The digit could not be actively flexed or extended by the patient but was passively correctable, consistent with Quinnell grade 3 (severe) triggering [13]. A comprehensive functional evaluation of the flexor tendons was performed. The flexor digitorum superficialis (FDS) was found to be non-functional, as evidenced by the inability to achieve isolated proximal interphalangeal joint (PIPJ) flexion during both the standard FDS test and the distal interphalangeal joint (DIPJ) extension test. The function of the flexor digitorum profundus (FDP) was also notably diminished, with active flexion at the DIPJ and overall finger flexion being significantly weaker and more restricted than in the contralateral, unaffected digits [20]. This profound functional impairment of both FDS and FDP provided a clinical correlate to the significant structural pathology later identified on imaging. Radiographs (Figure 1) demonstrated a bony ridge at the FDS insertion site on the middle phalanx.

**Figure 2:**
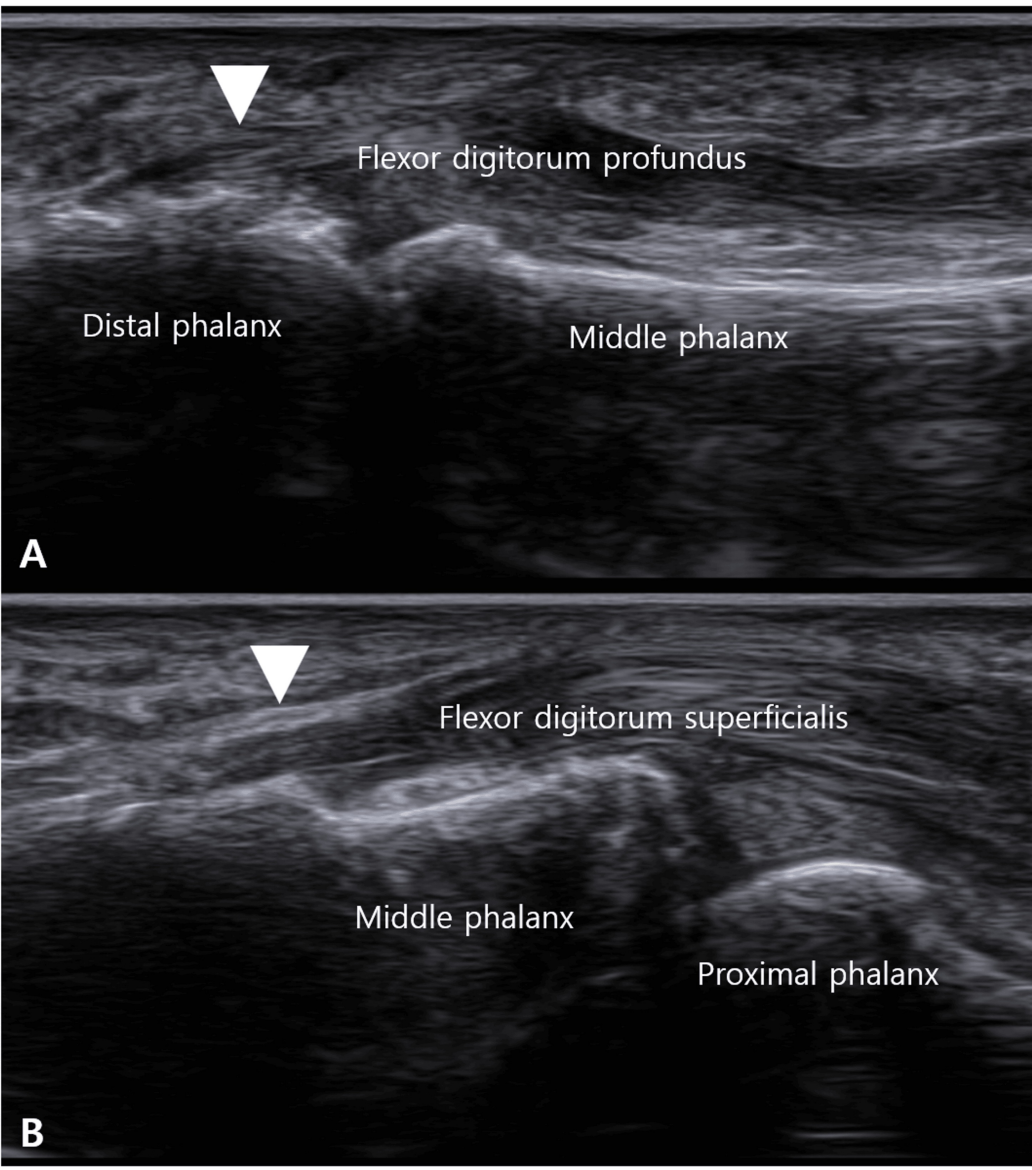
Ultrasound long-axis view showing palmar bony ridges (white arrowheads) at the attachment sites of the flexor digitorum profundus (A) and flexor digitorum superficialis (B) to the distal and middle phalanx.

Ultrasonography showed no significant pathology at the A1 pulley. However, it revealed calcification at the FDP insertion on the distal phalanx and calcification with hypoechoic thickening at the FDS insertion on the middle phalanx (Figure 2).

The diagnosis of insertional flexor tendinopathy as the primary cause of her triggering was confirmed by the triad of (1) radiographic evidence of a bony spur at the FDS, FDP insertion, (2) ultrasonographic findings of enthesopathy at both the FDS and FDP insertions, and (3) the clinical finding of restricted independent FDS, FDP function. Accordingly, the patient underwent two sessions of ultrasound-guided prolotherapy, targeting the pathological entheses of both the FDS and FDP tendons with a solution of 10% dextrose water (D10W) and 0.2% lidocaine. Following the second session, the patient’s triggering resolved completely, and her pain, rated on a visual analog scale, subsided from 7/10 at initial presentation to 1/10. This improvement was maintained throughout a three-month follow-up period.

## Discussion

This case report challenges the prevailing pulley-centric model of TF by demonstrating that the primary pathology can reside in the flexor tendon entheses rather than at the A1 pulley. Our patient’s refractory symptoms, coupled with the definitive imaging findings of insertional tendinopathy and the subsequent successful outcome with enthesis-targeted prolotherapy, collectively argue for the recognition of insertional flexor tendinopathy as a distinct etiological subtype of TF.

The traditional pathogenesis model of TF centers on a disproportion between the flexor tendon volume and the A1 pulley, leading to a constrictive tenosynovitis [13]. Consequently, diagnostic and therapeutic efforts are predominantly focused on this region. However, a growing body of evidence suggests that other structures, including the camper’s chiasm and the A0 (palmar aponeurosis) and A2 pulleys [6], can be implicated in persistent cases. Our findings extend this concept further, implicating the tendon-bone interface itself as a potential epicenter of pathology. The observed bony spurs on X-ray and the calcification with hypoechoic thickening on ultrasound are hallmark features of chronic enthesopathy, indicating a degenerative, rather than purely mechanical, process at play.

This insight provides a plausible explanation for cases refractory to conventional treatments. A CSI, while potent for reducing inflammatory tenosynovitis [21], may have limited and transient effects on a degenerative enthesophyte [22]. Similarly, an A1 pulley release addresses a potential site of secondary impingement but does nothing to treat the primary, diseased insertion point. We propose an analogy: managing TF solely by releasing the A1 pulley is akin to widening the tunnel to solve a problem caused by a train with a damaged and swollen carriage. The immediate obstruction may be relieved, but the fundamental fault in the train remains, predisposing it to future breakdowns elsewhere on the track.

This model may offer a plausible explanation for the clinical observation of some patients developing recurrent or multiple TFs, as the underlying tendon diathesis is not addressed [23]. This concept of a systemic tendon predisposition is further supported by the well-documented clinical association between TF and carpal tunnel syndrome [24,25]. Both conditions share common pathophysiological features of flexor tenosynovitis and frequently co-exist, particularly in patients with systemic risk factors such as diabetes mellitus [26]. The not uncommon development of TF following carpal tunnel release surgery [27] suggests that addressing one constrictive site does not eliminate the underlying biological tendency for tendon pathology. Therefore, we hypothesize that insertional flexor tendinopathy may represent another manifestation within this spectrum of flexor tendon pathology, extending beyond the traditional pulley-centric model.

Prolotherapy emerges as a rational and targeted intervention for this newly defined subtype. By injecting a proliferant such as hyperosmolar dextrose directly into the pathological enthesis, the treatment is thought to initiate a localized inflammatory response, which subsequently promotes fibroblast proliferation, collagen synthesis, and tissue remodeling, ultimately leading to tendon strengthening and repair [28]. This mechanism of action is fundamentally different from the anti-inflammatory effect of corticosteroids or the mechanical decompression of surgery. The success of prolotherapy in managing other enthesopathies, such as lateral epicondylitis and Achilles tendinopathy [29,30], provides a strong rationale for its use. Its application to insertional flexor tendinopathy represents a logical and promising extension of this regenerative approach.

While surgical A1 pulley release is highly effective for many, it is not without risk. Complications, including persistent pain, digital nerve injury, bowstringing, and CRPS, are well-documented in the literature [15,17]. Therefore, identifying a patient cohort that may benefit from a less invasive, biology-focused treatment such as prolotherapy is of significant clinical value. It offers a potential way to avoid surgical risks while directly addressing the pathological tissue.

Study limitations

The primary limitation of this study is its nature as a single case report. While the temporal relationship between prolotherapy and symptom resolution is compelling, it does not establish causality with the certainty of a controlled trial. Furthermore, the long-term durability of this treatment in such cases remains to be evaluated through studies with longer follow-up periods. Finally, the prevalence of this insertional tendinopathy subtype within the broader TF population is currently unknown and requires systematic radiographic and ultrasonographic screening in future prospective studies.

## Conclusions

This case demonstrates that insertional flexor tendinopathy can be a primary etiology of TF. A comprehensive ultrasound evaluation extending beyond the A1 pulley to the tendon entheses is crucial for accurate diagnosis. For patients with insertional pathology, enthesis-targeted treatments such as prolotherapy present a promising, minimally invasive therapeutic option that addresses the root cause and may prevent the need for surgery. Further research, including larger prospective cohort studies and randomized controlled trials comparing enthesis-targeted treatments to conventional pulley-centric approaches, is warranted to validate this tendon-centric model.
